# Amyloid Aggregation of Insulin: An Interaction Study of Green Tea Constituents

**DOI:** 10.1038/s41598-020-66033-6

**Published:** 2020-06-04

**Authors:** Miroslav Gancar, Elena Kurin, Zuzana Bednarikova, Jozef Marek, Pavel Mucaji, Milan Nagy, Zuzana Gazova

**Affiliations:** 1 Department of Biophysics, Institute of Experimental Physics, Slovak Academy of Sciences, Watsonova 47, 040 01 Kosice, Slovakia; 2 Department of Pharmacognosy and Botany, Faculty of Pharmacy, Comenius University in Bratislava, Odbojarov 10, 832 32 Bratislava, Slovakia

**Keywords:** Biophysics, Chemical biology

## Abstract

Exogenous insulin, used as a therapeutic agent for diabetes, forms insoluble deposits containing amyloid fibrillar structures near the administration site. We have analyzed the *in vitro* anti-amyloid activity of four green tea constituents: (-)-epigallocatechin gallate (EGCG), (-)-epicatechin (EC), gallic acid (GA), caffeine (CF), and their equimolar mixtures. Regarding individually tested compounds, only EGCG inhibited the fibrillization process. The individual EC, GA, and CF molecules were ineffective. The presence of EGCG in equimolar combinations with GA, EC, or CF was required for the inhibitory activity of most mixtures. Molecular docking revealed that EGCG interacts with an essential amyloidogenic region of insulin chain B. Individually inactive GA had a potentiating effect on the activity of EGCG. In contrast, EC and CF had a negative impact on the activity of the mixtures. We have observed diverse morphology and the amount of insulin amyloid aggregates formed in the presence of studied compounds. The distinct types of amyloid aggregates created *in vitro* in the presence of EGCG and other green tea constituents were characterized. Results indicate that the biological activity of individual molecules is not directly applicable to the pooled samples effects prediction.

## Introduction

Insulin is an important hormone acting as a regulator of carbohydrates and fat metabolism^1^. In its native state, insulin consists of 51 amino acids divided into two chains, which are connected by an intrachain and two interchain disulfide bonds and possess mostly an α-helical secondary conformation. However, its globular structure can be altered to a fibrillar form under different conditions through the manufacturing operations^2^, especially in association with the insulin pump used for diabetes therapy^3^. An occasional side effect is the formation of amyloid fibrils made of insoluble insulin deposits around the place of its application^4^, leading to the pathological condition known as injection amyloidosis^5^.

The initialization of amyloidogenesis is followed by an opening of the interior regions of the protein when hydrophobic amino acid residues are shifted to the surface of insulin. From this new position, they incline to the association and formation of oligomers, subsequently extending to the protofibrils and insoluble mature fibrils^3^. Insulin amyloids, similarly to other amyloid fibrils, possess typical structural characteristics, including diameter in 8–13 nm range and length up to several micrometers. They are arranged in a firm, unbranched structures, with organized β-strands ordered perpendicularly to the extended axis of the fibril. Understanding of the process of amyloid structures development would help to clarify the origin of amyloidoses^6^. Experimental *in vitro* formation of insulin fibrils may be a worthy and useful model in such research^1,7^.

Small natural molecules, abundant in medicinal plants, could be used as promising inhibitors of amyloid formation due to many advantages connected to their specific structure and stability in different types of body fluids^8^. The anti-aggregation activity of small compounds is based on their ability to form different types of bonds and/or interactions with amino acid residues (*e*.*g*., hydrogen bond, π-stacking or charge-to-charge complexes) possibly leading to a delay and/or an interruption of protein aggregation in different stages of fibrillization^9^. Many natural compounds can inhibit the amyloid fibril formation of human insulin in a dose-dependent manner^5,10^. For example, curcumin, ferulic acid, quercetin, and silibinin can inhibit the formation of the bovine insulin amyloids^11–13^. The study of Malisauskas *et al*. explored the data from 265 flavones and discovered that the presence of hydroxyl group at specific positions 5, 6, 7, and 4’ and its concurrent absence at position 3 is an important factor in the inhibition of human insulin amyloid formation^14^.

Green tea, more specifically the infusion from the dry leaves of *Camellia sinensis* (L.) O. Kuntze plant is known to be a healthy beverage with strong antioxidant potency. It is rich in polyphenols, among them catechins, (-)-epigallocatechin gallate (EGCG), flavonols, their glycosides, and depsides. Caffeine is also a typical constituent at a usual level of 3%^15^. Different human studies claim that green tea may be helpful at the reduction of cardiovascular disease risk, and some forms of cancer, improvement of oral health and body weight control^16^. Daily drinking of green tea in large doses is associated with a reduced prevalence of cognitive impairment in elderly Japanese people^17^. It has an impact on the extracellular deposition of the amyloid-β peptide, hyperphosphorylation of tau protein, and can affect basal mechanisms of Alzheimer’s disease^18^. Catechins in green tea extracts diminish toxicity induced by amyloid-β-derived peptides in rat hippocampal cell cultures. EGCG and gallic acid reduce amyloid-β aggregation and formation of Aβ-derived neurotoxin ligands^19^.

Moreover, gallic acid inhibits the conformational conversion of α-helix to β-sheet^20^. EGCG delays the insulin secondary structure transformation and formation of amyloid fibrils^7^. Many proteins, e.g., α- and β-casein, amyloid-β, α-synuclein, and islet amyloid polypeptide, interact with EGCG non-covalently and non-specifically, which affects their physiological function. Hydrogen bonding and hydrophobic interactions seem to be important mechanisms of EGCG action due to its eight hydroxyl groups on three aromatic rings^7^.

However, the effect of the whole extract cannot be described by a straightforward interpretation of its single constituents due to their mutual interactions. Moreover, green tea polyphenols can act synergistically with a different tendency in many models. For example, in a model describing the prevention against the formation of advanced glycation end-products can gallic acid synergistically inhibit the cross-β structure formation and protein carbonyl content in fructose-glycated BSA in the presence of ascorbic acid^21^. Green tea catechins (mostly EGCG) mixed with albumin can synergistically increase its antioxidant activity in oil-water emulsion by the formation of protein-catechin adducts^22^. Synergy was also observed between green tea extract and EGCG, as well as quercetin and EGCG, by an *in vitro* antioxidant assay^23^. Effects of EGCG and A-type EGCG dimer on insulin fibril formation were studied^24^ as well as their impact on other proteins and peptides^25–29^. In antimicrobial studies, epicatechin shows synergy with theaflavins against isolated clinical samples of *Stenotrophomonas maltophilia* and *Acinetobacter baumannii*^30^. Green tea catechins exhibited a synergistic effect when accompanied by fluconazole and amphotericin B against *Candida glabrata* and *Candida albicans*^31^.

There has not been published any interaction study regarding green tea polyphenols and amyloid-forming proteins. In our previous work, we confirmed the synergistic effects of red wine polyphenols mixtures on inhibition of the lysozyme amyloid formation and the destruction of amyloid fibrils^32^.

In our present work, we focus on the inhibition of insulin amyloid aggregation with typical green tea constituents and the impact of their mutual interactions on their activity. Since we cannot predict the effects of individual substances, an inclusive, experimental exploration of the interactions among green tea polyphenols is essential. This study brings us closer to the understanding of common co-operation of plant extracts and substances in nature. It might help us interpret the dynamic relationships of single compounds in mixtures.

## Results and Discussion

**Figure 1:**
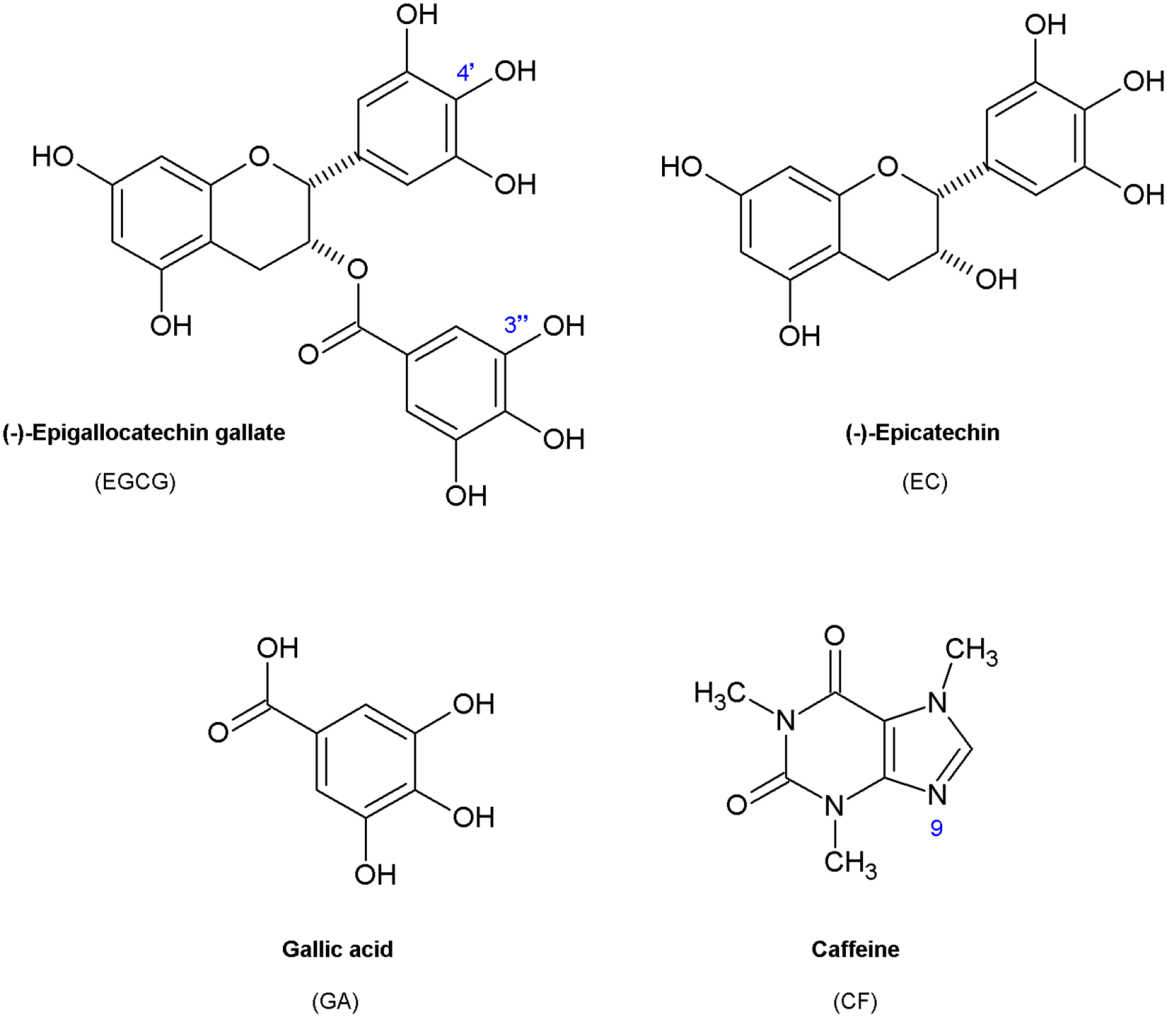
Structures of studied green tea constituents.

The goal of this study was to investigate the effects of the green tea constituents (Fig. 1) and their equimolar mixtures (Table 1) on the formation of amyloid fibrils derived from human insulin.

**Table 1:** Individual green tea constituents and their equimolar mixtures.

Individual compounds	Binary mixtures	Ternary mixtures	Quaternary mixture
GA EGCG EC CF	GA:EGCG GA:EC GA:CF EGCG:EC EGCG:CF EC:CF	GA:EGCG:EC EGCG:EC:CF GA:EGCG:CF GA:EC:CF	GA:EGCG:EC:CF

### The effect of compounds and equimolar mixtures on amyloid fibrillization of human insulin

**Figure 2:**
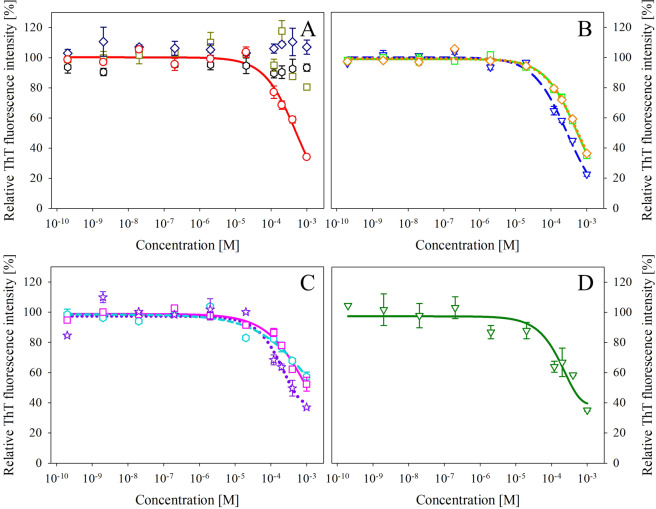
ThT fluorescence intensities determined for amyloid aggregation of insulin in the presence of an increasing concentration of (**A**) solo compounds – GA (black hexes), EC (dark yellow squares), EGCG (red circles) and CF (dark blue diamonds); (**B**) EGCG containing binary mixtures – GA:EGCG (blue triangles); EGCG:EC (light green squares); EGCG:CF (orange diamonds) or (**C**) ternary mixtures – GA:EGCG:CF (turquoise hexagons); GA:EGCG:EC (magenta squares); EGCG:EC:CF (purple stars) and (**D**) quaternary mixture – GA:EGCG:EC:CF (dark green triangles). The data represent the average fluorescence value of three independent measurements (n = 3) with an average deviation.

The effect of studied compounds and their mixtures on the amyloid aggregation of human insulin was investigated using the ThT fluorescence assay. The formation of amyloid aggregates is accompanied by an increase of ThT fluorescence intensity as a result of ThT binding to the cross-β sheet structure of amyloids. Thus, the decrease in ThT fluorescence intensity represents the inhibitory activity of studied compounds on the amyloid aggregation of insulin. Individual compounds or their equimolar mixtures were added in the specified concentration range to insulin solutions to evaluate their dose-response effect. The obtained average data were normalized to the ThT signal of untreated insulin amyloid fibrils (Fig. 2). Resulting fluorescence values were fitted, and IC_50_ values (corresponding to a concentration of compound/mixture with 50% inhibitory activity) were calculated and are presented in Table 2.

**Table 2:** IC_50_ values, related standard deviations of studied compounds, and their equimolar mixtures.

Individual compounds	IC_50_ [µM]	Binary mixtures	IC_50_ [µM]
GA EGCG EC CF	N/A 476.1 ± 32.5 N/A N/A	GA:EGCG GA:EC GA:CF EGCG:EC EGCG:CF EC:CF	268.6 ± 40.8 N/A N/A 514.8 ± 13.0 525.9 ± 32.0 N/A
**Ternary mixtures**	**IC**_**50**_ **[µM]**	**Quaternary mixture**	**IC**_**50**_ **[µM]**
GA:EGCG:EC EGCG:EC:CF GA:EGCG:CF GA:EC:CF	1032.7 ± 85.8 350.5 ± 24.8 1753.6 ± 33.6 1332.8 ± 59.9	GA:EGCG:EC:CF	390.4 ± 24.2

*N/A stands for “not available”.

Regarding individual compounds, EGCG was the only one possessing inhibiting properties as a substantial decrease in ThT fluorescence, starting at 100 μM compound concentration was observed (Fig. 2A, red circles). The calculated IC_50_ value is equal to 476.1 μM. Other tested compounds GA (Fig. 2A, black hexes), EC (Fig. 2A, dark yellow squares), and CF (Fig. 2A, blue diamonds) did not inhibit the fibrillization process within studied concentration range.

Out of 6 binary equimolar mixtures, only ones containing EGCG had a significant effect on the amyloid aggregation of insulin. Mixture GA:EGCG (Fig. 2B, blue triangles) had the lowest IC_50_ value out of all studied combinations equal to 268.6 μM, suggesting that non-active GA potentiate observed moderate activity of EGCG alone. Mixtures EGCG:EC (Fig. 2B, light green squares) and EGCG:CF (Fig. 2B, orange diamonds) displayed slightly diminished inhibitory effect compared to EGCG alone with higher IC_50_ values equal to 514.8 μM and 525.9 μM, respectively. Binary mixtures not containing EGCG did not exhibit an inhibitory effect within the studied concentration range (Fig. S1A).

Ternary mixtures inhibited the amyloid aggregation of insulin in different extent (Figs. 2C and S1B). EGCG remained the fundamental compound as the most potent ternary mixture was the EC:EGCG:CF (Fig. 2C, purple stars). Mixtures containing GA - GA:EGCG:EC (Fig. 2C, magenta squares), GA:EGCG:CF (Fig. 2C, turquoise hexes) and GA:EC:CF (Fig. S1B blue diamonds) had lower or no potency to affect the insulin fibrils formation. Determined IC_50_ values increase in the following order: EGCG:EC:CF < GA:EGCG:EC < GA:EC:CF < GA:EGCG:CF and are equal to 350.5 μM, 1032.7 μM, 1332.8 μM, and 1753.6 μM, respectively. Interestingly, ternary mixtures containing GA and EGCG in combination with either EC or CF had a weaker effect than binary mixture GA:EGCG. On the other hand, mixture EGCG:EC:CF displayed better efficiency in comparison to binary mixtures EGCG:EC, EGCG:CF, and also EGCG alone.

The quaternary mixture GA: EGCG: EC: CF (Fig. 2D, dark green triangles) exhibited inhibiting properties similar to the ternary EC: EGCG: CF mixture with IC_50_ value equal to 390.4 μM. All IC_50_ values are summarized in Table 2.

### Kinetic study of insulin amyloid aggregation – an influence of green tea constituents

**Figure 3:**
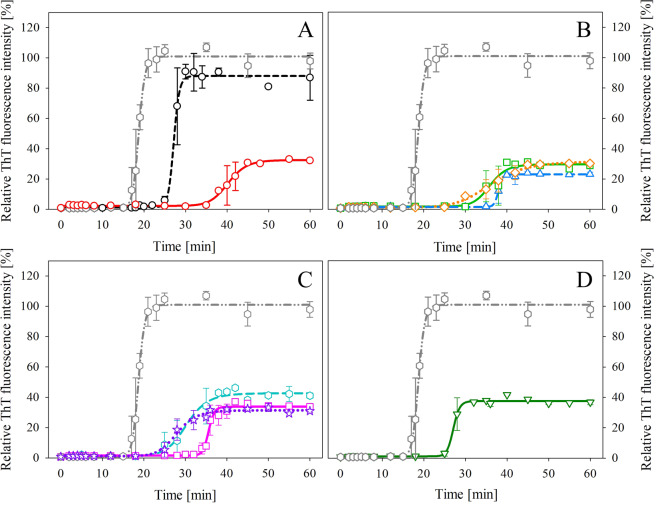
Kinetic profiles of insulin amyloid aggregation without treatment (dark grey hexes in **A**–**D**) and in the presence of (**A**) solo compounds – GA (black circles) and EGCG (red circles); (**B**) binary mixtures – GA:EGCG (blue triangles), EC:EGCG (light green squares) and CF:EGCG (orange prisms); (**C**) ternary mixtures – GA:EGCG:EC (magenta squares), GA:EGCG:CF (cyan hexes), and EC:EGCG:CF (purple stars) or (**D**) quaternary mixture – GA:EC:EGCG:CF (dark green triangles). The error bars represent the average deviation of three separate measurements (n = 3). The concentration of the insulin was 20 µM, and compound concentration was 1 mM.

To clarify the inhibitory effect of studied compounds, we investigated the kinetics of insulin amyloid aggregation by ThT fluorescence assay. The aggregation followed a nucleation-elongation model expected for amyloid assembly represented by a sigmoidal profile of growth curves^33^. The growth curve of untreated human insulin (Fig. 3A–D – dark grey hexes) is composed of a so-called lag phase represented by the *t*_*lag*_ parameter equal to ~17 min. During this phase, nuclei formation occurs due to intermolecular interactions of non-native monomeric protein species and their subsequent oligomerization^34^. The lag phase is followed by an elongation or growth phase, associated with polymerization of oligomers into amyloid fibrils. The elongation phase of insulin is characterized by parameters *t*_*half*_ ~ 18 min and *k*_*agg*_ ~ 1.36 min^−1^. The last stage, called plateau or equilibrium phase, is characterized by the presence of mature amyloid aggregates. The kinetic parameters are summarized in Table 3.

**Table 3:** Kinetic parameters derived from aggregation kinetics of insulin alone or in the presence of studied compounds and their equimolar mixtures at 1 mM concentration. *t*_*lag*_ denotes the duration of the lag phase, *t*_*half*_ denotes the halftime of the aggregation, *k*_*agg*_ stands for aggregation constant, and R is the correlation coefficient. Every experiment was performed as a triplicate (n = 3), and final data represent an average value accompanied by a corresponding standard deviation.

Sample	*t*_*lag*_ [min]	*t*_*half*_ [min]	*k*_*agg*_ [min^−1^]	R
Insulin	17.26 ± 0.16	18.73 ± 0.07	1.36 ± 0.15	0.998
GA	25.83 ± 0.45	27.17 ± 0.20	1.49 ± 0.30	0.998
EGCG	35.91 ± 0.46	40.30 ± 0.26	0.46 ± 0.05	0.996
GA:EGCG	36.97 ± 0.35	38.03 ± 0.05	1.89 ± 0.64	0.999
EGCG:EC	31.97 ± 1.20	36.06 ± 0.67	0.49 ± 0.10	0.984
EGCG:CF	27.90 ± 0.78	36.19 ± 0.42	0.24 ± 0.02	0.997
GA:EGCG:EC	34.37 ± 0.54	35.59 ± 0.13	1.64 ± 0.78	0.993
EGCG:EC:CF	23.22 ± 0.80	28.08 ± 0,44	0.41 ± 0.07	0.995
GA:EGCG:CF	25.95 ± 0.64	27.29 ± 0.25	1.49 ± 0.46	0.997
GA:EGCG:EC:CF	25.66 ± 0.45	27.13 ± 0.20	1.36 ± 0.27	0.998

Upon the addition of compounds and their mixtures, we observed various alternations in kinetic parameters. Solo compounds GA and EGCG (Fig. 3A, GA - black circles, and EGCG - red circles) both prolong the lag phase, although EGCG had a substantially higher effect with *t*_*lag*_ ~ 36 min compared to *t*_*lag*_ ~ 26 min in case of GA. Additionally, EGCG also exhibited an effect on aggregation constant (*k*_*agg*_ ~ 0.46 min^−1^) compared to GA (*k*_*agg*_ ~ 1.49 min^−1^), which is within a margin of error in comparison to the aggregation of insulin alone (*k*_*agg*_ ~ 1.36 min^−1^). Based on these results, together with measurements assessing the concentration-dependent effect, we suggest that EGCG is the only active individual compound.

Binary mixtures EGCG:EC and EGCG:CF (Fig. 3B - light green squares and orange prisms) both affected aggregation constant (*k*_*agg*_ ~ 0.49 min^−1^ and *k*_*agg*_ ~ 0.24 min^−1^, respectively). Mixture EGCG:CF significantly shortened the lag phase with *t*_*lag*_ ~ 28 min as opposed to *t*_*lag*_ ~ 32 min in the case of EGCG:EC. Mixture GA:EGCG (Fig. 3B - blue triangles) exhibited the most noticeable effect on the lag phase elongation (*t*_*lag*_ ~ 37 min), which translated into the best IC_50_ value (268.6 µM), but did not affect the aggregation constant (*k*_*agg*_ ~ 1.89 min^−1^)_._

Regarding ternary mixtures, GA:EGCG:EC (Fig. 3C - magenta squares) extended lag phase (*t*_*lag*_ ~ 34 min), but exhibited high aggregation constant (*k*_*agg*_ ~ 1.64 min^−1^) and low activity (IC_50_ = 1032.7 µM). Mixture GA:EGCG:CF (Fig. 3C - cyan hexes) affected the length of the lag phase (*t*_*lag*_ ~ 26 min); however, it did not influence the aggregation constant (*k*_*agg*_ ~ 1.49 min^−1^) and exhibited very low efficiency (IC_50_ = 1753.6 µM). On the other hand, in the case of mixture EGCG:EC:CF (Fig. 3C - purple stars) we observed much lower IC_50_ value (350.5 µM) and low aggregation constant (*k*_*agg*_ ~ 0.41 min^−1^), although the slightly lower impact on the length of the lag phase (*t*_*lag*_ ~ 23 min).

Quaternary mixture GA:EGCG:EC:CF (Fig. 3D - dark green triangles) affected the lag phase (*t*_*lag*_ ~ 26 min), displayed reasonable IC_50_ value (390.4 µM), yet did not influence the aggregation constant (*k*_*agg*_ ~ 1.36 min^−1^).

All kinetic parameters obtained for insulin fibrillization in the absence and presence of studied compounds and their mixtures are summarized in Table 3. Collected data point out that we have mostly observed an effect arising from the presence of GA (high *k*_*agg*_) and EGCG (high *t*_*lag*_) or their adduct - the product of direct addition of two or more distinct molecules, resulting in a single reaction product^35^. The presence of other stable adducts (not only in equimolar ratios), which would explain the efficiency of all mixtures containing ECGC and GA, is discussed thoroughly in the part *Interactions analysis - suggested mechanism of action*.

### Morphology of amyloid aggregates

**Figure 4:**
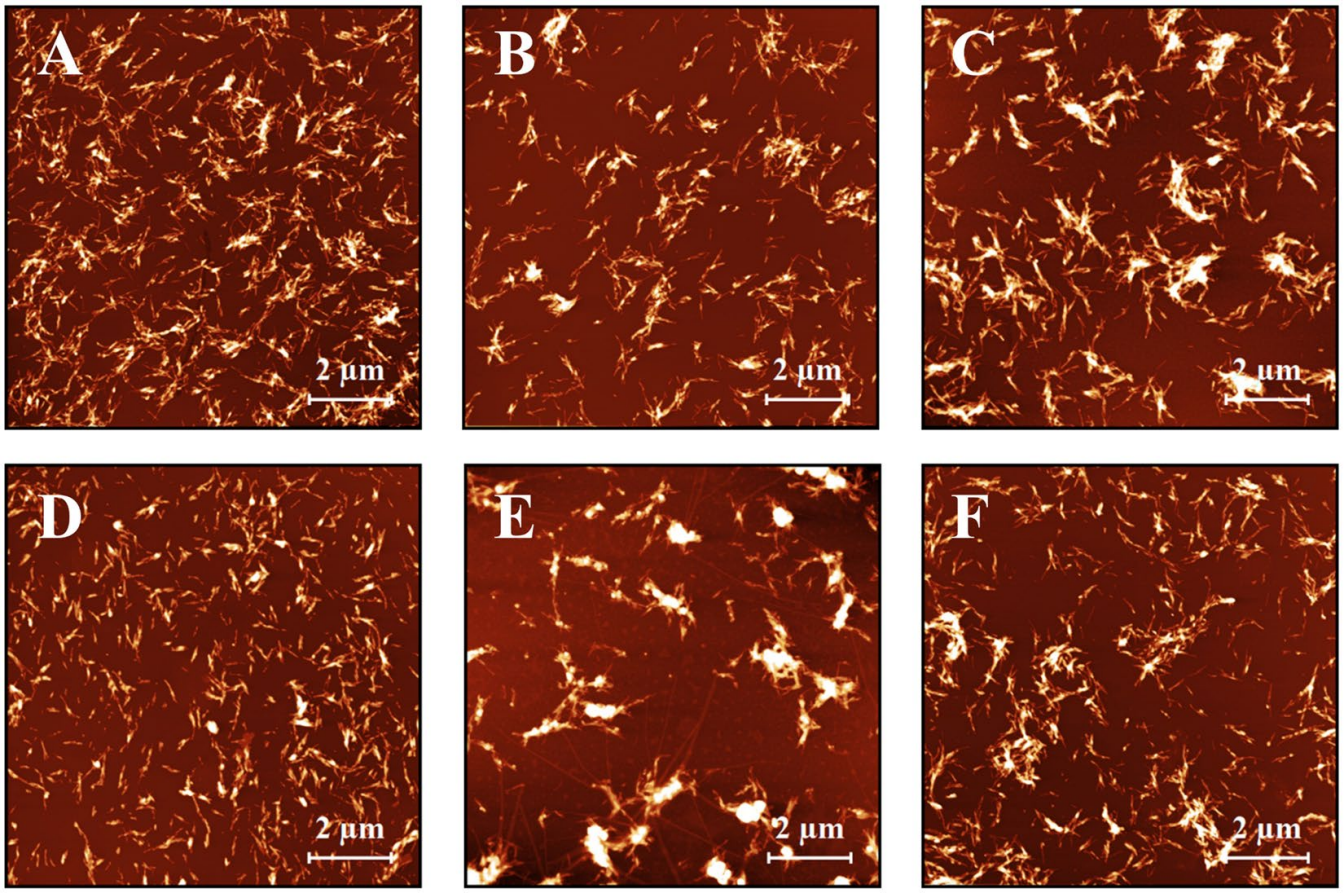
(**A**) AFM images of insulin amyloid fibrils (20 μM) formed alone or in the presence of (**B**) EGCG, (**C**) GA:EGCG, (**D**) EGCG:EC, (**E**) EGCG:CF and (**F**) GA:EGCG:EC:CF. The concentration of EGCG and mixtures was 1 mM. The xy scale of all images is 10 μm × 10 μm.

The AFM was used to verify the formation of insulin amyloid fibrils as well as for examination of the compounds’ ability to affect the quantity and morphology of the fibrils (Fig. 4). Exposure of solution of insulin monomers (20 μM) to an aggregation inducing conditions resulted in the formation of a high amount of amyloid fibrils (Fig. 4A). The addition of 1 mM EGCG led to a substantial decrease in the number of aggregates (Fig. 4B). Mixture GA:EGCG (Fig. 4C) promoted the formation of bigger stacks of aggregates (~10–70 nm high) (Fig. S2). Treatment of insulin fibrillization with mixture EGCG:EC led to the formation of very short, fragmented amyloid aggregates (Fig. 4D). The different effect was observed for insulin aggregation in the presence of mixture EGCG:CF (Figs. 4E and S3). The sample contained three different objects: very thin, ~3–5 nm, and few microns long fibrillar aggregates (Fig. S3A), over 80 nm tall aggregate bundles (Fig. S3B) as well as ~15–60 nm high stacked fibrils (Fig. S3C). The ternary mixture GA:EGCG:EC produced two different types of objects, namely the ~60–85 nm high stacked amyloid fibrils (Fig. S4A) and short, fragmented amyloid aggregates (Fig. S4B). For another ternary mixture, EGCG:EC:CF (Fig. S5A) only ~60–85 nm high stacked amyloid fibrils (Fig. S5B) were observed. Presence of last active ternary mixture GA:EGCG:CF (Fig. S6B) leads to the production of stacked amyloid fibrils, which were ~6–30 nm high (Fig. S6A). The quaternary mixture GA:EGCG:EC:CF (Fig. 4F) caused the formation of fibrils with a tendency to generate ~10–70 nm high lateral stacks (Fig. S7), which is comparable to the effect of GA:EGCG.

### Secondary structure determination – ATR-FTIR spectroscopy

**Figure 5:**
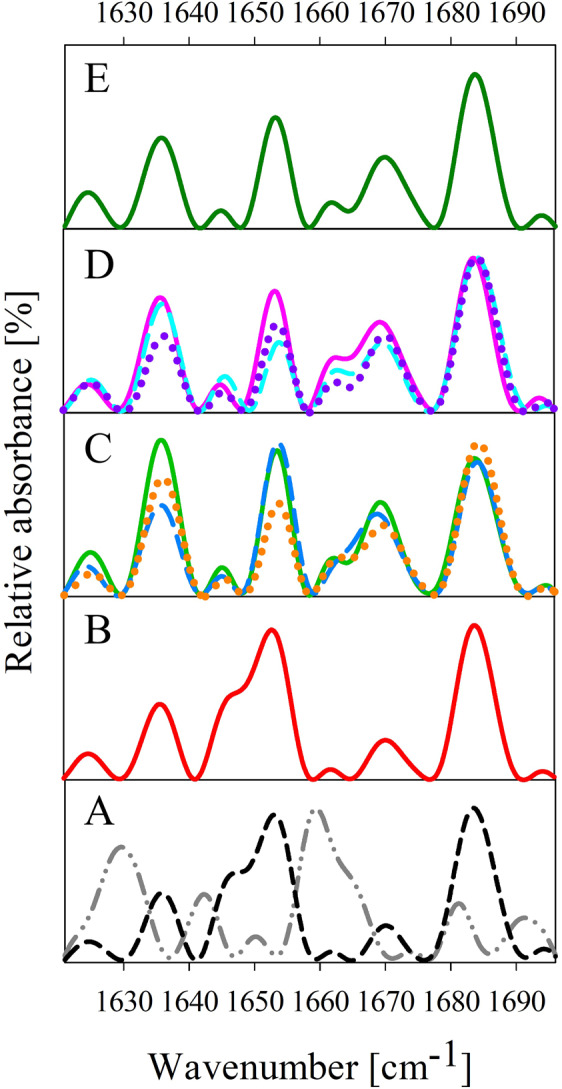
(**A**) ATR-FTIR spectra of 35 μM native insulin (black dashed line) and 35 μM insulin amyloid fibrils after fibrillization without added compound (dark gray dash-dot-dot line) or (**B**) in the presence of 1750 μM compound EGCG (red solid line); (**C**) in the presence of binary mixtures – GA:EGCG (light blue dashed line), EGCG:EC (green solid line), and EGCG:CF (orange dotted line); (**D**) in the presence of ternary mixtures – GA:EGCG:EC (magenta dotted line), GA:EGCG:CF (purple solid line), and EGCG:EC:CF (turquoise dashed line); and in the presence of ternary mixture GA:EGCG:EC:CF (green solid line). The resulting spectra represent an average of 128 repetitions.

The changes in the secondary structure of insulin were examined using ATR-FTIR spectroscopy. The IR spectra recorded for native insulin, insulin amyloid fibrils formed alone or in the presence of effective compound EGCG or EGCG-containing mixtures are presented in Fig. 5. Spectrum for mixture GA:EC:CF is shown in Fig. S8. The spectra were deconvolved (Fig. S9) to calculate the content of particular protein secondary structures (Table S1).

The absorption spectrum of native insulin in the amide I region (Fig. 5A, black dashed line) shows a wide band at 1654–1664 cm^−1^, corresponding to ~29% α-helical content. Bands at 1624–1643 cm^−1^ and 1689–1698 cm^−1^ represent ~17% β-sheet content. Contrary, insulin amyloid fibrils formed alone (Fig. 5A, dark gray dash-dot-dot line) show a significant increase in β-sheet content, characteristic for amyloid aggregates. Calculated β-sheet content for insulin amyloid fibrils was ~48%. This significant increase was mainly at the expense of random coil, β-turn, and α-helical content compared to the native insulin at used experimental conditions.

Upon the addition of EGCG (Fig. 5B, red line), we have observed almost perfect preservation of secondary structure compared to the native insulin. All binary mixtures (Fig. 5C) affected the secondary structures of aggregates in similar manner. Mixture GA:EGCG (Fig. 5C, light blue dashed line) conserved most of the α-helical content (~26%) and prevented an extensive β-sheet formation (~22%). On the other hand, aggregates formed in the presence of the mixture EGCG:EC (Fig. 5C, green solid line) contained ~31% β-sheet content mostly at the expense of β-turn content as this mixture preserved ~23% α-helical content. The aggregates formed in the presence of mixture EGCG:CF (Fig. 5C, orange dotted line) contained only ~20% of the α-helical structure and ~28% β-sheet secondary structure.

Ternary mixtures performed fairly similarly, as they inhibited the formation of β-sheet structure in the following order: EGCG:EC:CF (~22%) (Fig. 5D, purple dotted line) > GA:EGCG:CF (~25%) (Fig. 5D, turquoise dashed line) > GA:EC:CF (~26%) (Fig. S8, blue line) > GA:EGCG:EC (~27%) (Fig. 5D, magenta solid line), which correlates well with our results from ThT experiment.

Even though the presence of quaternary mixture GA:EGCG:EC:CF resulted in ~26% β-sheet formation, it also preserved ~22% of α-helical content (Fig. 5E).

The above mentioned data suggest that EGCG and studied mixtures affect the amyloid aggregation of human insulin to some extent. Interestingly, various mixtures of EGCG and green tea constituents potentiate the formation of β-turns associated with the loss of the random coil structure, while the particular aggregates differ in the β-sheet and preserved α-helix content.

### Molecular docking study

To explain observed (in)activity of tested compounds, we have performed *in silico* calculations used public docking web service. We have searched for interaction sites of individual substances on the monomeric insulin, which would theoretically explain: (a) the activity of sole EGCG, (b) inactivity of individual EC, GA, and CF, and (c) changes in activities of equimolar mixtures. Since the simultaneous docking service of two or more molecules to the protein is not available, we have compared the effect of the position of each ligand on the monomeric insulin (always an analysis of 10 best docking poses) to the (in)activity of the respective mixtures. To simplify this process, we have assumed that the decisive step in the insulin fibrillization seems to be its dimer formation. This approach has allowed us to explain: activity of sole EGCG, inactivity of sole EC, GA, and CF, as well as the negative impact of EC or CF on IC_50_ values, respectively, and the positive influence of GA in binary mixtures containing EGCG.

**Figure 6:**
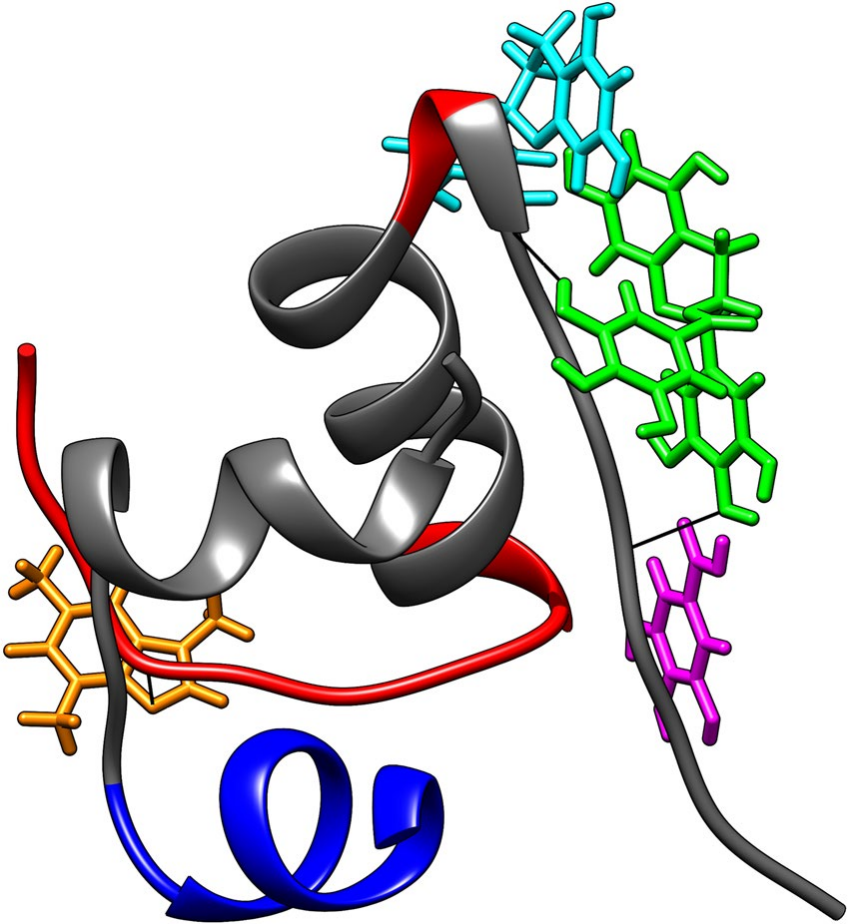
Structure of human insulin at pH 1.9 (PDB ID: 2MVC) and best docking poses for studied compounds (GA - magenta; EGCG - green; EC - turquoise; CF - ochre) Calculated amyloidogenic regions are colored in grey. Molecular graphics image was produced using the UCSF Chimera 1.13 package (Resource for Biocomputing, Visualization, and Informatics at the University of California, San Francisco, CA, USA, https://www.cgl.ucsf.edu/chimera/).

At first, we have calculated amyloidogenic regions of monomeric insulin: from Ile10 to Asn21 in the chain A, and from Leu11 to Cys19 and from Arg22 to Thr30 in the chain B, respectively (Fig. 6, grey sections of human insulin chains). EGCG is the only tested ligand that interacts with the essential B-chain amyloidogenic region of insulin (Arg22-Tyr26). This interaction is stabilized through two hydrogen bonds: (1) between the oxygen of 4′-OH group of EGCG and the hydrogen of amide bond of Tyr26 with Phe25 (2.49 Å), and (2) between the hydrogen of 3″-OH group of EGCG and the oxygen of carbonyl group of Arg22 (2.07 Å) (Fig. 6, green). GA also binds to insulin in another amyloidogenic region (Tyr26-Pro28), but this interaction is apparently not critical for preventing putative insulin dimers formation (Fig. 6, magenta). EC only interacts with a small part of the amyloidogenic region (Tyr16-Cys19) (Fig. 6, turquoise). Molecular docking showed that CF interacts far away from all amyloidogenic areas (Fig. 6, ochre) and thus, does not influence the EGCG binding behavior. This interaction is stabilized through the hydrogen bond between N9 of caffeine and the hydrogen of the amide bond situated between His5 and Gln4 (2.26 Å).

### Interactions analysis - suggested mechanism of action

As we have shown, the effect of solo compounds on insulin fibrils formation can be explained by binding positions of individual compounds with insulin monomer. However, the reason behind the effect of their equimolar mixtures is more complex. Starting with binary mixtures, GA can act synergistically and promote the effect of EGCG as both amyloidogenic regions of insulin are blocked simultaneously by EGCG and GA, respectively. Therefore, resulting mutual IC_50_ is lower than the one observed for the sole EGCG. One could also consider the interaction of insulin with the GA:EGCG 1:1 adduct, which has already been described in the crystalline form^36^ and analyzed by using the Quantum Theory of Atoms in Molecules^37^, but its presence or stability in (acidic) solution is not confirmed. Moreover, the insulin lag phase in the presence of GA:EGCG mixture is comparable to solo EGCG, indicating instability of GA:EGCG adduct in acidic conditions. Since only one product of the fibrillization process (stacked fibrils) was detected, one can presume either free EGCG (active) and GA (inactive) are present from the beginning of this experiment or GA:EGCG 1:1 adduct has decomposed in an acidic environment almost immediately at the beginning of the experiment, and no concurrent active species (EGCG and GA:EGCG) were present during the incubation.

In the case of EGCG:EC mixture, the free EGCG is partially displaced from the ideal amyloidogenic site by the interaction with EC. As a result, the activity of this binary mixture deteriorates compared to the sole EGCG. Another possible explanation is the effect of EGCG:EC adduct (1:1), the existence of which was confirmed only in a neutral aqueous solution^38^, but its presence at pH = 1.6 is not known. Considering only one product of fibrillization process was produced - short and fragmented amyloid aggregates, one can assume that observed inhibitory effect is either due to presence of free EGCG (active) and EC (inactive) or EGCG:EC adduct has decomposed almost immediately at the beginning of the experiment as a consequence of an acidic environment, and no two concurrent active species (EGCG and EGCG:EC) were present. Different products of fibrillization processes with GA:EGCG and EGCG:EC (stacked fibrils *vs*. very short, fragmented amyloid aggregates) confirm the distinct influence of GA and EC, respectively, on EGCG interaction with insulin.

The binary mixture EGCG:CF is active, though its activity was worse than individual EGCG. However, the presence of three different adducts EGCG:CF (1:1, 2:2, and 2:4) should be considered^39,40^. Moreover, the stability study of different adducts with CF, including EGCG or EC, showed that they are breaking down slowly in 3% acetic acid creating free CF, EGCG, or EC^23^. Our kinetic results further support these studies since we have observed varying *t*_*half*_ values (Table 2). Since EGCG:CF mixture was prepared in DMSO, possibly other analogous adducts mentioned above were present immediately after adding the sample to the insulin solution at pH = 1.6. During the 120 minutes incubation, the amount of free CF and EGCG could arise and interact with insulin. As mentioned above (*Morphology of amyloid aggregates*), three different objects were observed, which implicates implicates three different, and concurrent fibrillization processes have taken place due to three different ligands/adducts present in the reaction mixture. If EGCG:CF adduct 1:1, 2:2, or both were present at the beginning of the experiment, they could create a mixture of CF, EGCG, and EGCG:CF in an acidic solvent. As individual CF is not active, three active species would be present: EGCG, EGCG:CF 1:1, and EGCG:CF 2:2. Theoretically, another option is possible too. EGCG and CF mixed in the equimolar ratio (e.g., 4:4) can create adduct EGCG:CF 2:4 with remaining free two parts of active EGCG. The EGCG:CF 2:4 adduct (may be active one) could break down in starting mixture in an acidic environment to a 1:1 or 2:2 adduct (each of them may be active) and free CF. Then, after some time, both adducts (1:1 or 2:2) could create additional free CF and additional free (active) EGCG. In total, at an unspecified time during 120 minutes incubation, three active species (EGCG:CF 2:4, EGCG:CF 1:1 or 2:2, and EGCG) are present in the reaction mixture. In summary, both proposed scenarios fit with the arise of three different fibrillization products. As IC_50_ of EGCG:CF sample is higher than one of EGCG, it can be considered that EGCG:CF adducts 1:1 and 2:2 are less active than sole EGCG.

Ternary mixture GA:EGCG:EC consists of one active component (EGCG) and two inactive ones (GA and EC). It could be formally considered as an “overlap” of binary combinations GA:EC (inactive), GA:EGCG, and EGCG:EC (both active). The justification for GA:EGCG and EGCG:EC mixtures mentioned above can be used since the GA:EGCG:EC mixture produces both analogous fibrillization products (stacked fibrils and very short, fragmented amyloid aggregates) and similar derived kinetic parameters (Table 3). We suggest that the increase in IC_50_ value is the result of a negative contribution of GA:EC and EGCG:EC to the overall activity of the ternary mixture.

Regarding mixture EGCG:EC:CF, the observed stacked amyloid fibrils (Fig. S5B) are analogous to ones from the EGCG:CF experiment. Theoretically, the prepared ternary mixture contains mainly EC: CF and EGCG:CF species at the beginning of the experiment as their association constants with CF are significantly higher (577.2 M^−1^ and 940.7 M^−1^ at 313 K, respectively) compared to EC:EGCG (13.4 M^−1^ at 300 K)^38,39^. Thus, the entire amount of CF (e.g., 2 μM) would be bound in equally (e.g., 1 μM) to EC and EGCG, respectively. This process results in the presence of unbound EC (inactive) and EGCG (active), always at 1 μM concentration. Besides, decomposition of CF:EGCG in acidic solvent leads to yet another free EGCG explaining notably lower IC_50_ value of ternary mixture compared to the original CF:EGCG mixture (where more EGCG is bound to CF at the beginning of the experiment). We have concluded that EGCG is the only active individual compound. Therefore, we propose this adduct stability order under applied experimental conditions: CF:EGCG > CF:EC > EC:EGCG > GA:EGCG, assuming that longer *t*_*lag*_ corresponds to the lower stability of the adduct. While it corresponds well with the above-mentioned association constants, it also supports the considered instability of GA:EGCG adduct.

Presence of the ternary mixture GA:EGCG:CF leads to the production of stacked amyloid fibrils (Fig. S6B). Analogous objects were also detected in GA:EGCG and CF:EGCG experiments. These adducts are theoretically present together with GA:CF observed in this experiment. Its existence as 1:1 adduct in neutral water was recently described^41^. However, it was not effective in an acidic environment in our study. Since the association constant of GA:CF adduct is unknown, presumption given in the previous paragraph is not applicable now. Moreover, markedly increased IC_50_ value for GA:EGCG:CF compared to one for sole EGCG suggests that CF preferably forms an adduct with EGCG, which is in correlation with the proposed adduct stability order. This process causes a significant decrease of free EGCG amount in comparison to the CF:EGCG or GA:EGCG experiments.

Only one type of insulin amyloid aggregates (stacked fibrils) was detected in the presence of GA:EC:EGCG:CF (Fig. S7). Summarizing arguments from previous experiments, one can assume that CF preferentially creates adducts with EGCG and EC, and to a smaller extent, with GA. As the quaternary mixture was prepared in an equimolar ratio, real reaction conditions could recall the case of ternary mixture EC:EGCG:CF, including better IC_50_ value compared to individual EGCG.

The most common types of amyloid aggregates observed in our experiments are stacked fibrils of different sizes (6–85 nm). They are formed by the action of almost all tested mixtures containing EGCG. Results from the AFM morphology study correlate well with our ATR-FTIR experiments since we observed a rather similar secondary structure content for all tested mixtures. The only exception is the mixture EC:EGCG, which produces only very short, fragmented amyloid aggregates, probably due to the slight steric interaction between parent compounds in the amyloidogenic region. Interestingly, the ATR-FTIR spectrum for this mixture showed that its presence resulted in the formation of the highest amount of aggregates with the highest β-sheet content amongst all tested mixtures, indicating a higher quantity of amyloid fibrils as opposed to amorphous aggregates. We suggest that variability in the size of stacked fibrils and other observed aggregates is related to the concurrent actions of various adducts and individual compounds.

## Conclusions

In conclusion, we have explored the effects of four compounds naturally present in green tea leaves ((-)-epigallocatechin gallate, (-)-epicatechin, gallic acid, and caffeine) and their equimolar mixtures on inhibition of insulin amyloid aggregation. Our results show that only (-)-epigallocatechin gallate acts as a sole active constituent. Each of the mixtures containing EGCG possesses an anti-amyloid activity suggesting the intrinsic activity of EGCG is not lost by the contribution of compounds. However, their presence leads to different efficiencies depending on the ability of co-compounds to create variable adducts among themselves and their binding activity to amyloidogenic regions. Using atomic force microscopy, we found out that different ECGC-containing mixtures lead to the formation of not-identical types of fibrils. Our research confirms the need to explore the impact of small molecule mixtures on the final anti-amyloid activity as the contribution of other molecules in extracts can influence the final mixture’s effect even if the activity of the single substance is known.

## Materials and Methods

### Chemicals

Human insulin (I2643, recombinant, expressed in yeast, CAS number: 11061-68-0), Thioflavin T (ThT), dimethyl sulfoxide (DMSO), sodium chloride (NaCl), hydrochloric acid (HCl), and green tea constituents (Fig. 1, Table 1): gallic acid (GA), (-)-epigallocatechin gallate (EGCG), (-)-epicatechin (EC), caffeine (CF) were purchased from the Sigma Aldrich Chemicals Company (St Louis, MO). All chemicals were of analytical reagent grade. All possible equimolar mixtures (Table 1) of all four tested compounds were dissolved in DMSO.

### *In vitro* insulin amyloid aggregation

Insulin was dissolved in ultrapure water with pH adjusted to 1.6 by the addition of HCl and diluted to a final 20 µM concentration. Subsequently, NaCl was added (50 mM) to finalize the protein stock solution. The insulin solution was incubated at 50 °C for 120 min under constant stirring at 1200 rpm in a thermomixer. The formation of insulin amyloid aggregates was monitored by a Thioflavin T (ThT) fluorescence assay and atomic force microscopy.

### ThT fluorescence assay

The presence of insulin amyloid fibrils is associated with an intensive increase of ThT fluorescence intensity. ThT was added to samples (ratio ThT:protein = 2:1) after the aggregation process up to a final concentration of 40 µM, and samples were incubated at 37 °C for 45 min. The ThT fluorescence intensity was measured by Synergy Mx microplate reader with the excitation set to 440 nm and the emission recorded at 485 nm (6 mm offset).

### Inhibition of insulin amyloid aggregation, determination of IC_50_ values

The effect of studied compounds and their equimolar mixtures on the amyloid aggregation of insulin was examined by ThT fluorescence assay. Experiments were carried out at the fixed 20 µM insulin concentration. Compounds were dissolved in DMSO and added to insulin solutions in a concentration ranging from 200 pM to 1 mM keeping the volume of DMSO lower than 2% at all times. Sample solutions were exposed to amyloid aggregation inducing conditions, as described above. Measured ThT fluorescence intensities were normalized to the ThT fluorescence signal of insulin amyloid aggregates alone. Each experiment was performed as a triplicate, and the final fluorescence value represents the average of 3 separate measurements, with corresponding errors included. The IC_50_ values (concentration of compound/mixture with 50% inhibitory activity) were determined from curves obtained by fitting the average ThT fluorescence intensities by non-linear least-squares method (Equation: Sigmoid, Parameter 4; Hill 3) in the SigmaPlot 12.0 software (Systat Software Inc., USA).

### Kinetics of amyloid aggregation and analysis of kinetic data

The ThT assay was used to measure the kinetics of untreated insulin amyloid aggregation (20 µM) or treated with 1 mM concentration of individual compounds and their mixtures. The samples were incubated at amyloid inducing conditions mentioned above and withdrawn at given time intervals. Every experiment was performed as a triplicate, the resulting data represent average values, and the error bars denote the average deviation.

The kinetics of insulin aggregation was represented by changes in the ThT fluorescence intensity plotted as a function of time. Experimental data were subsequently analyzed by nonlinear regression using a standard sigmoidal described by Eq. (1):

1 $$y={A}_{1}+({A}_{2}-{A}_{1})/(1+exp(-{k}_{agg}(t-{t}_{half})))$$

where $y$ is the fluorescence intensity, ${A}_{1}$ and ${A}_{2}$ are the initial and the final values of fluorescence intensity, ${k}_{agg}$ is the apparent fibril growth rate, $t$ is the time and ${t}_{half}$ stands for the time at half-height of fluorescence maximum. Equation 1 was modified by relation ${k}_{agg}=\,2/({t}_{half}-{t}_{lag})$ and subsequently used to fit experimental data to determine the lag time parameter^42^.

### Atomic force microscopy

Insulin sample solutions were applied on a surface of freshly cleaved mica. After 5 min adsorption, the surface of mica was rinsed several times by ultrapure water and left to dry. AFM images were obtained by Scanning Probe Microscope (Veeco di Innova) in a tapping mode using SNL-10 (silicone tip on nitride lever coated with 4 nm Ti/Au layer) cantilever. All images are unfiltered and processed in Gwyddion 2.50.

### Attenuated total reflectance Fourier transform infrared (ATR-FTIR) spectroscopy

Attenuated total reflectance FTIR (ATR-FTIR) spectra were recorded using Nicolet^TM^ 8700 Fourier transform infrared spectrometer (Thermo Fisher Scientific) equipped with Smart OMNI-Sampler (diamond crystal). 5 µl of a sample (35 µM human insulin, 1750 μM compound) was spread on the diamond surface. Every sample was measured as a triplicate. Each spectrum represents an average of 128 repetitions recorded at the resolution of 2 cm^−1^ in the amide I region (1700–1600 cm^−1^). Recorded spectra were smoothed using OMNIC 8 software (Thermo Fisher Scientific) to achieve the quality of spectra adequate for deconvolution. 11-point Savitzky–Golay filter (10.607 cm^−1^) followed by 7-point Savitzky–Golay filter (6.750 cm^−1^) was applied. Spectra were subsequently deconvoluted by the peak analyzer in OriginPro 8 (OriginLab Corporation) (Fig. S8). Baseline was subtracted, and the positions of peaks in the amide I region were added manually in correlation with raw data. To correctly assign peak positions to the secondary structures, measured spectra were compared against the published literature^43^. Gaussian peak function was used to fit the data, and appropriate secondary structures content was obtained by integration of resulting curves.

### Molecular docking

The human insulin monomeric structure in solution at pH = 1.9 from the Protein Data Bank (PDB ID: 2MVC) was used for calculations. Three-dimensional structures of EGCG, EC, GA and CF (ligands) were created in ChemSketch software (ChemSketch, version 12.01, Advanced Chemistry Development, Inc., Toronto, ON, Canada) and converted from a *.mol file format to a *.mol2 one by OpenBabel 2.3.2 and used without any optimization. SwissDock web server (http://www.swissdock.ch/docking#) was used to dock monomeric insulin with all ligands separately. The best 10 docking solutions were evaluated for all ligands. Molecular graphics images were produced using the UCSF Chimera 1.13 package (Resource for Biocomputing, Visualization, and Informatics at the University of California, San Francisco, CA, USA). Hydrogen bonds were calculated using relax constraints of 0.4 Å and 20.0 degrees, respectively. Amyloidogenic regions of insulin monomer were calculated by WALTZ software (http://waltz.switchlab.org), threshold = high sensitivity, and pH = 2.6.

## Supplementary information

Supplementary information.

## Data Availability

The datasets generated during and/or analyzed during the current study are available from the corresponding author on reasonable request.
