# Supplementary material for: Amyloid Aggregation of Insulin: An Interaction Study of Green Tea Constituents
Source: Sci Rep. 2020 Jun 4;10:9115. doi: 10.1038/s41598-020-66033-6 (PMC7272432; doi:10.1038/s41598-020-66033-6)
Supplement: Supplementary file 1 — Supplementary information. [file 41598_2020_66033_MOESM1_ESM.docx]

**Supplementary information**

**Title:** Amyloid Aggregation of Insulin: An Interaction Study of Green Tea Constituents

**Authors:**

Miroslav Gancar^a^, Elena Kurin^b*^, Zuzana Bednarikova^a^, Jozef Marek^a^_,_ Pavel Mucaji^b^, Milan Nagy^b^, Zuzana Gazova^a,*^

**Author Affiliation:**

^a^Department of Biophysics, Institute of Experimental Physics Slovak Academy of Sciences, Watsonova 47, 040 01, Kosice, Slovakia

^b^Department of Pharmacognosy and Botany, Faculty of Pharmacy, Comenius University in Bratislava, Odbojarov 10, 832 32, Bratislava, Slovakia

**Corresponding Authors (^*^):**

Zuzana Gazova, Department of Biophysics, Institute of Experimental Physics Slovak Academy of Sciences, Watsonova 47, 040 01, Kosice, Slovakia, [gazova@saske.sk](mailto:gazova@saske.sk), Tel: +421 (55) 7204135

Elena Kurin, Department of Pharmacognosy and Botany, Faculty of Pharmacy, Comenius University in Bratislava, Odbojarov 10, 832 32, Bratislava, Slovakia, [elena.kurin@uniba.sk](mailto:elena.kurin@uniba.sk), Tel: +421 (2) 50117170

**Figure S1:** ThT fluorescence intensities determined for amyloid aggregation of insulin in the presence of a different concentration of non-effective binary mixtures GA:EC (cyan blue circles), GA:CF (green squares), EC:CF (dark violet triangles) and only effective ternary mixture free of EGCG, GA:EC:CF (IC_50_ = 1332.8 μM) (blue diamonds).

A B


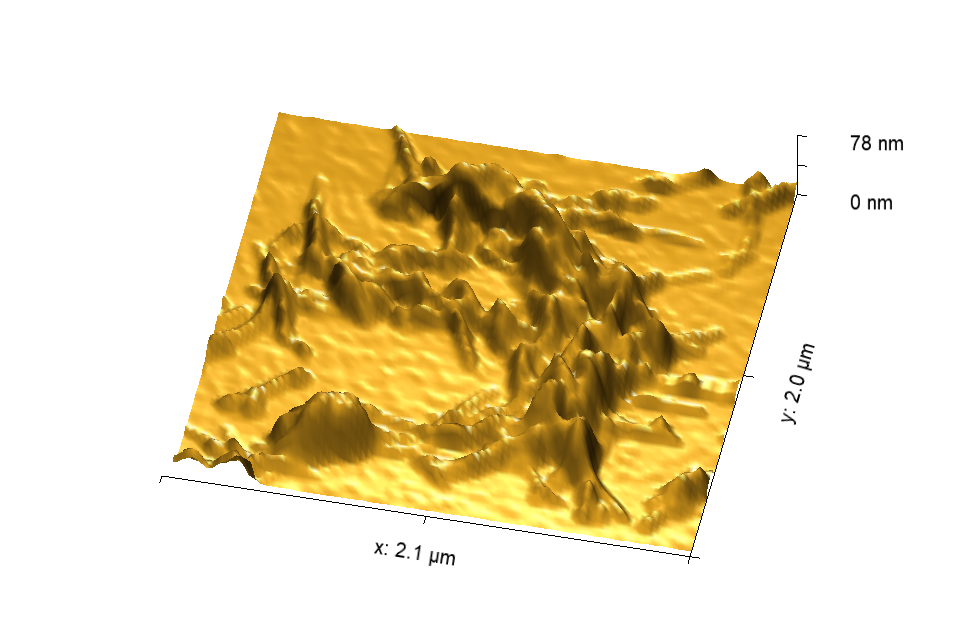
**Figure S2:** 3D visualization of insulin amyloid aggregates in the presence of mixture GA:EGCG. 3D images were produced in Gwyddion 2.50.


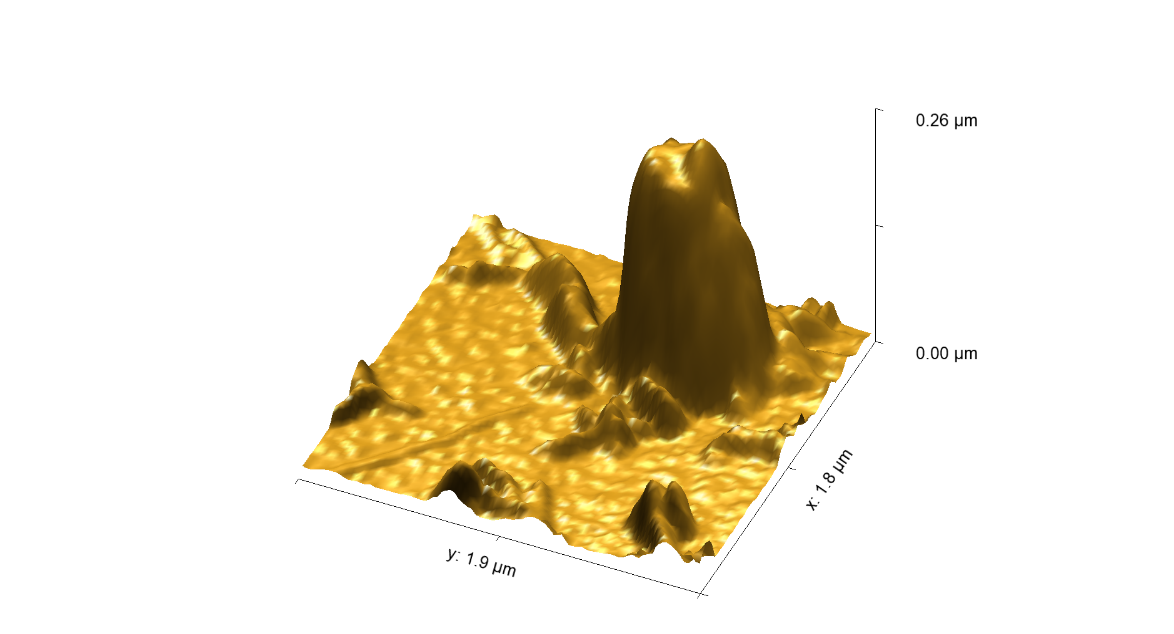

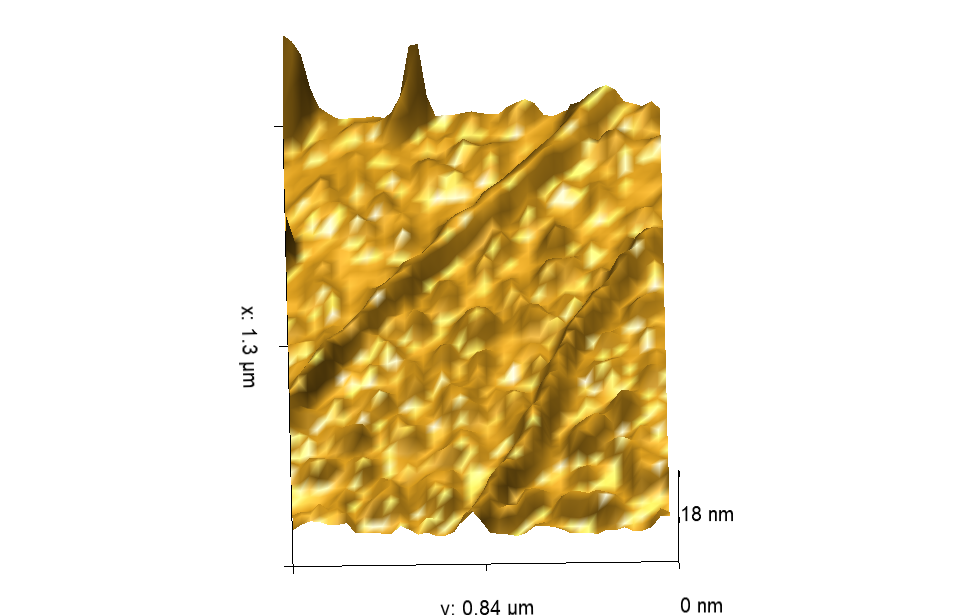

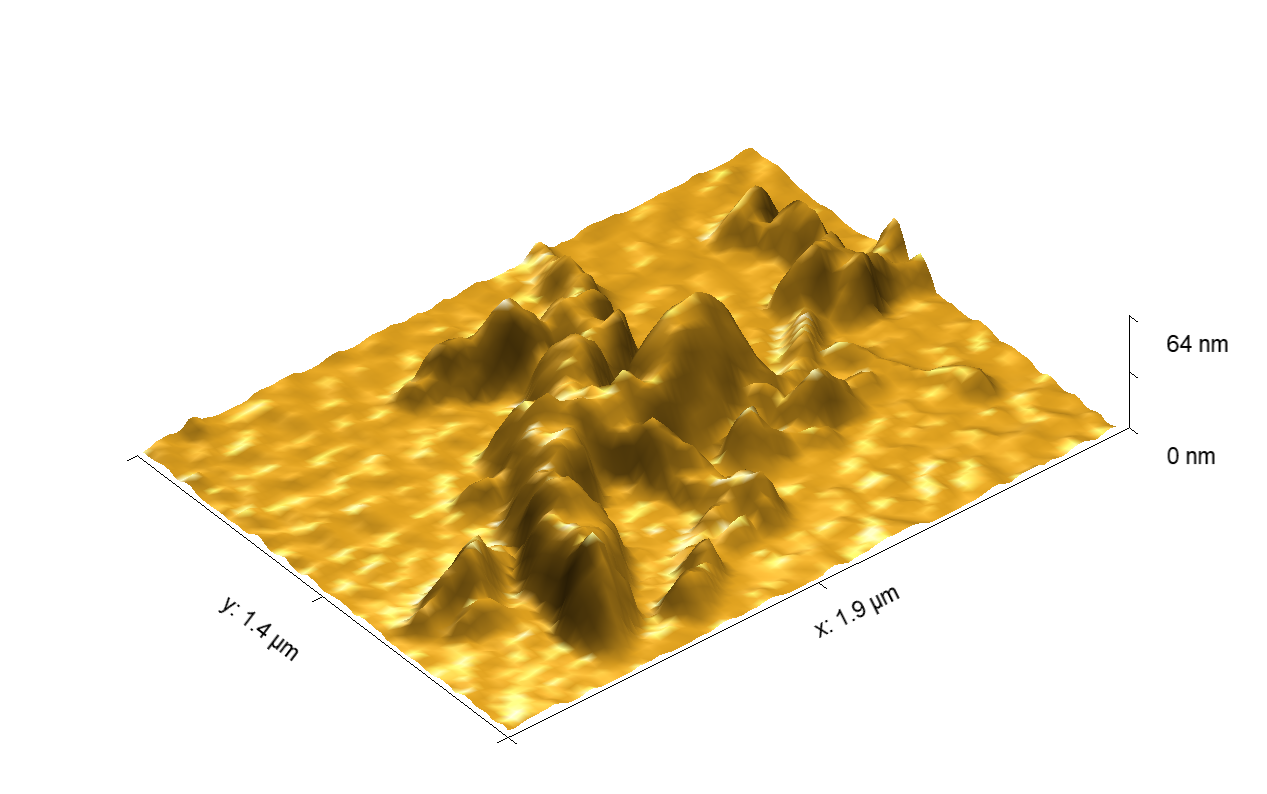


**A B**

**C**

**Figure S3:** Three different insulin amyloid aggregates formed in the presence of mixture EGCG:CF. (A) very thin and few microns long fibrils; (B) high aggregate bundles, and (C) stacked amyloid fibrils.


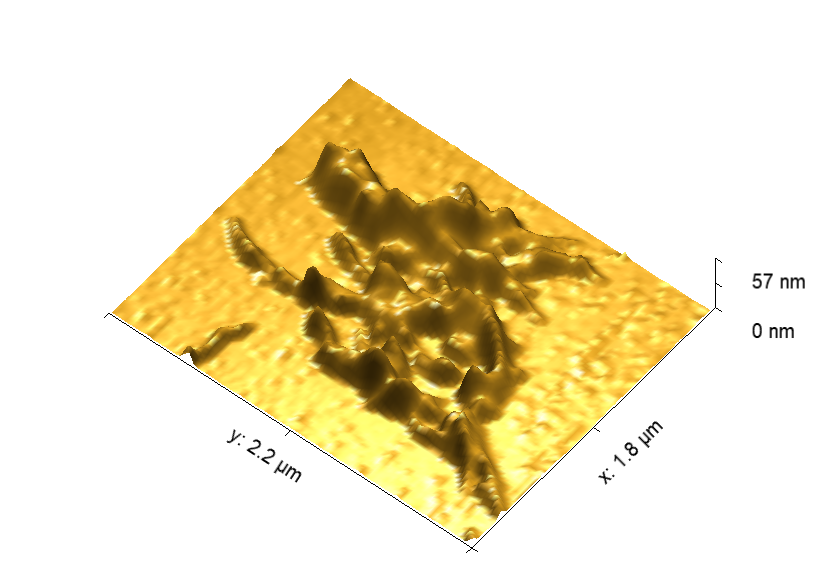

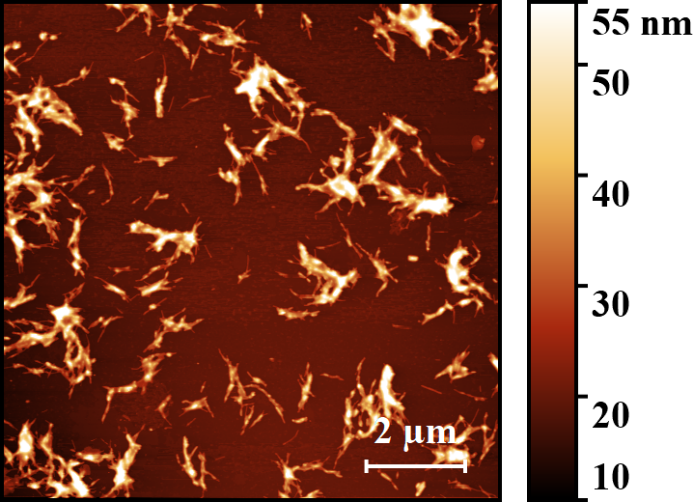


**A B**

**Figure S4:** (A) 3D visualization of stacked insulin amyloid fibrils and (B) AFM image of aggregates formed in the presence of ternary mixture GA:EGCG:EC.

**Figure S5**: (A) 3D visualization of stacked insulin amyloid fibrils and (B) AFM image of aggregates formed in the presence of ternary mixture EGCG:EC:CF.


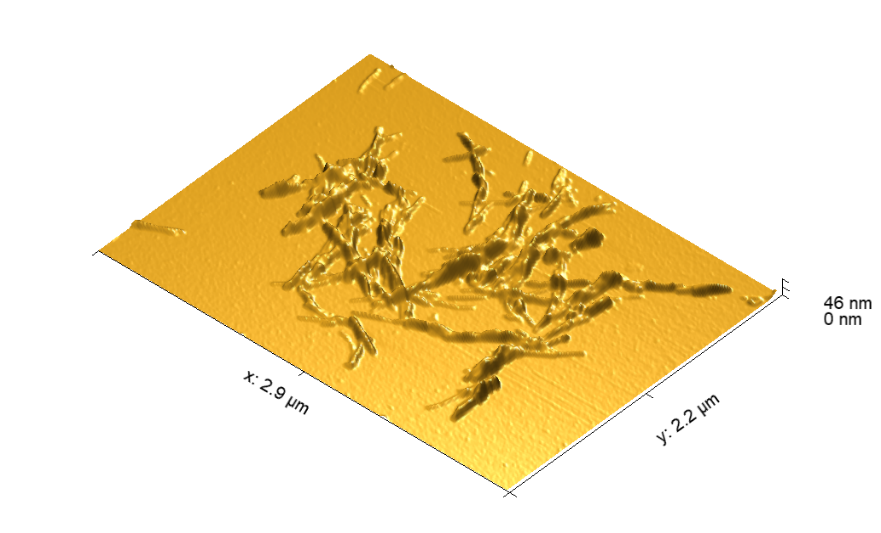

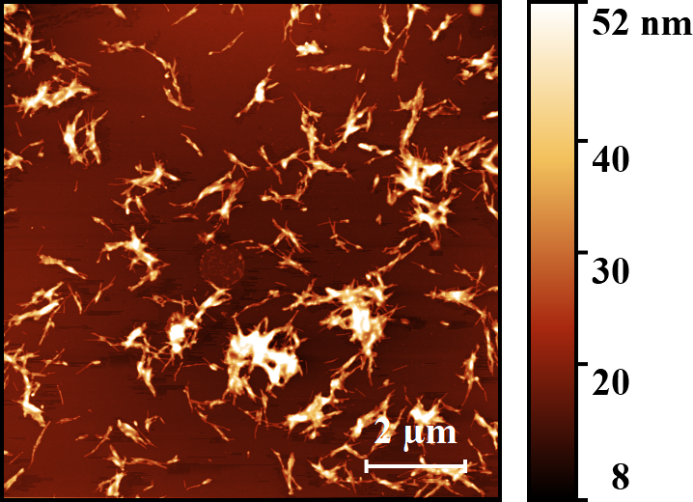


**A B**


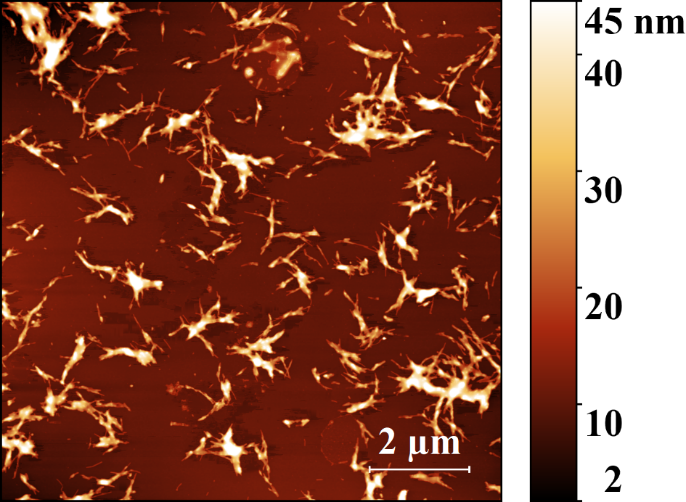

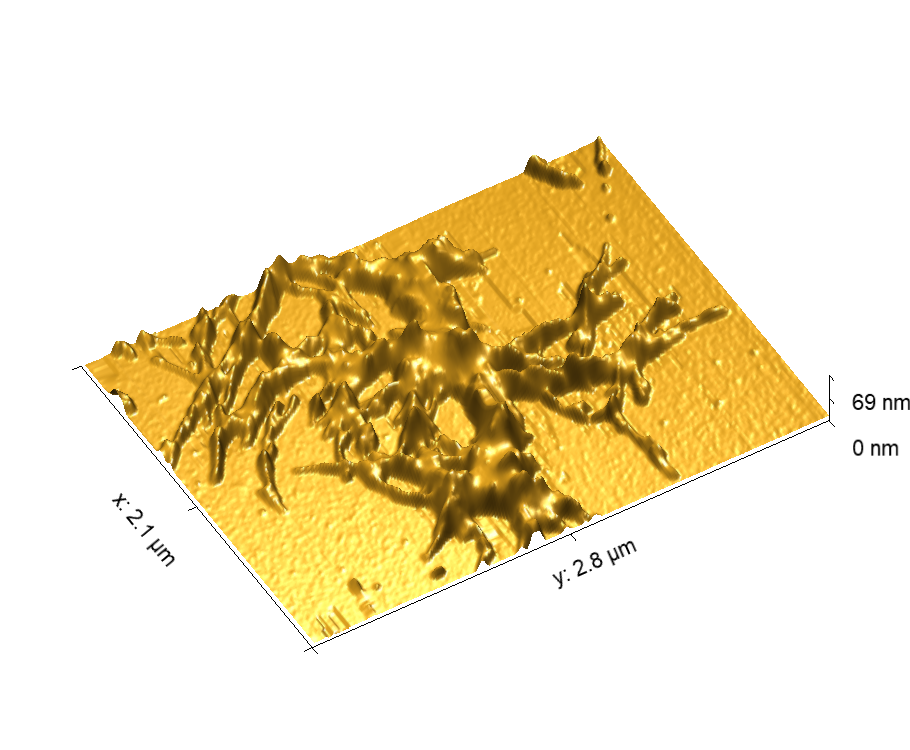


**A B**


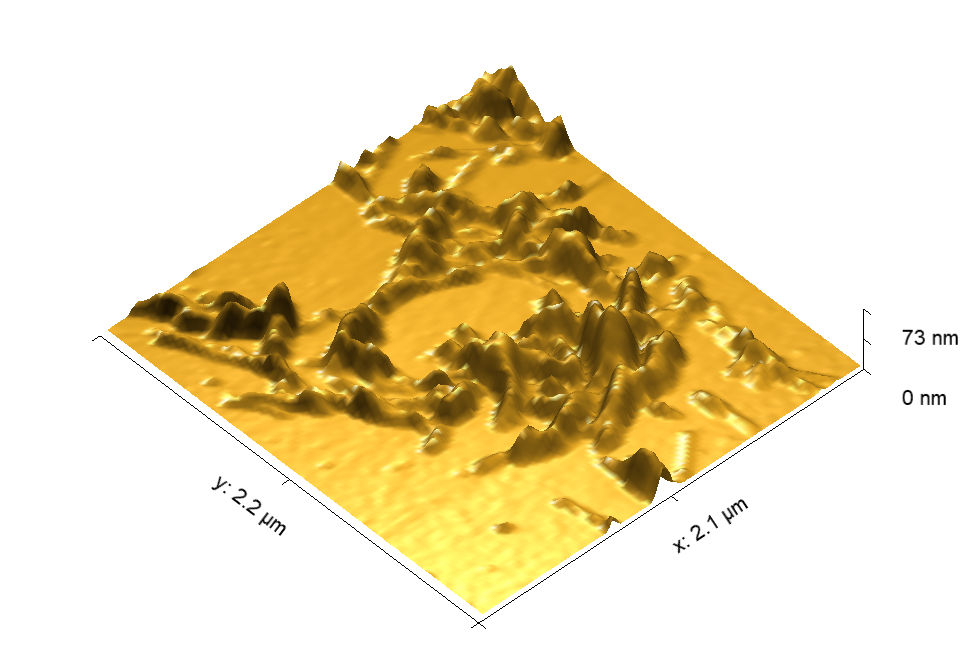
**Figure S6:** (A) 3D visualization of stacked insulin amyloid fibrils and (B) AFM image of aggregates formed in the presence of ternary mixture GA:EGCG:CF.

**Figure S7:** 3D visualization of insulin amyloid aggregates in the presence of mixture GA:EGCG:EC:CF.


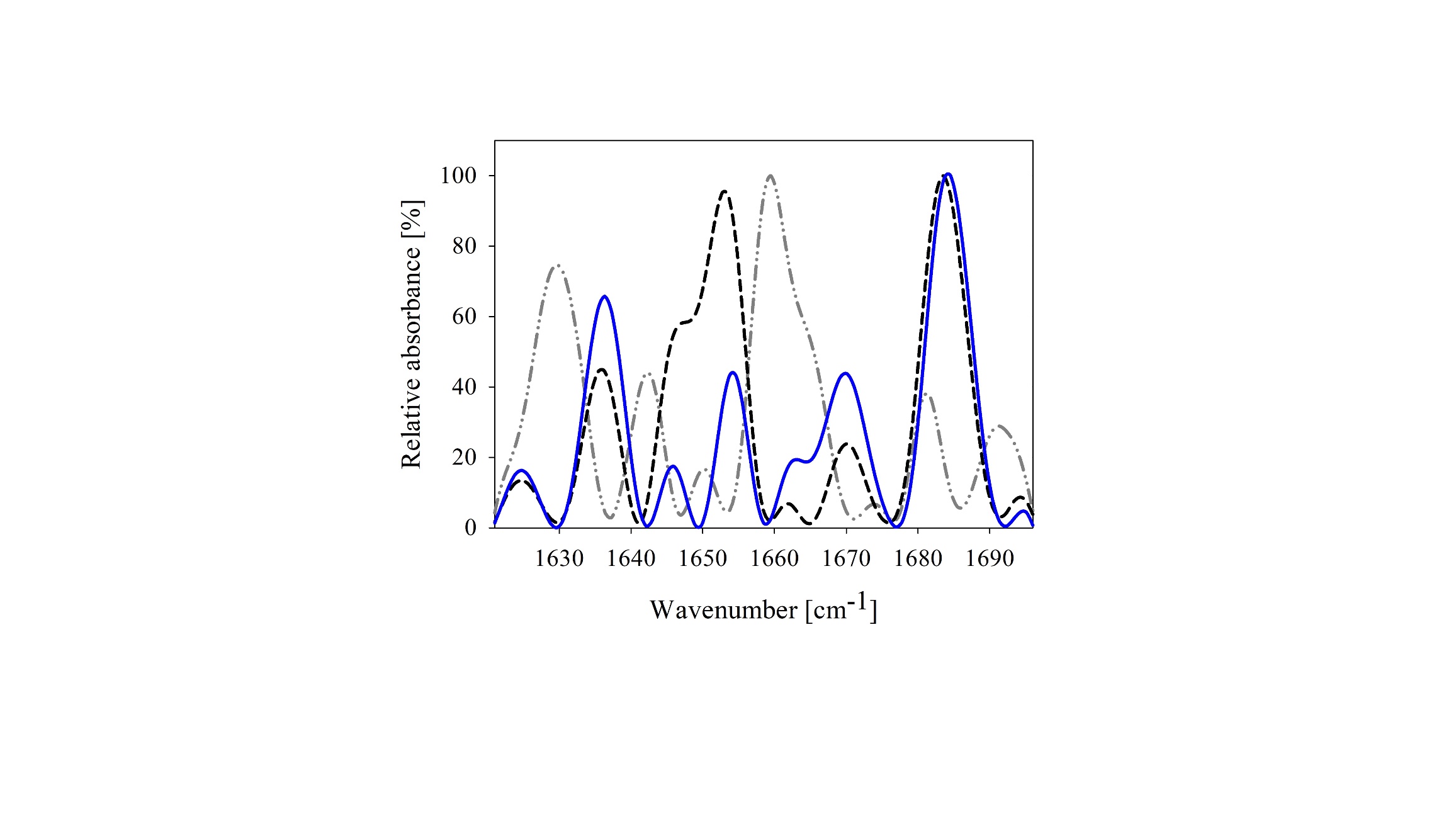
**Figure S8:** ATR-FTIR spectra of 35 μM native insulin (black dashed line), 35 μM insulin amyloid fibrils formed alone (dark gray dash-dot-dot line), and in the presence of 1750 μM ternary mixture GA:EC:CF (blue line).


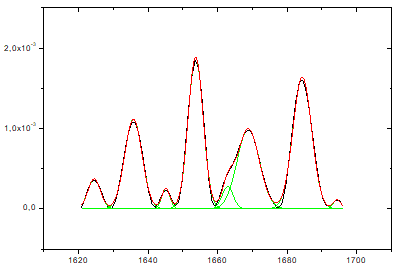


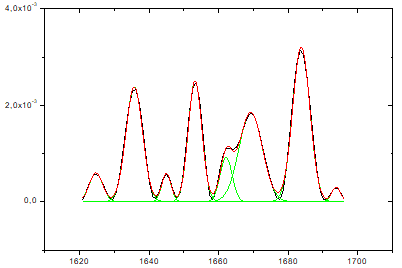

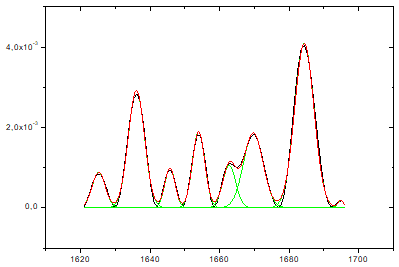

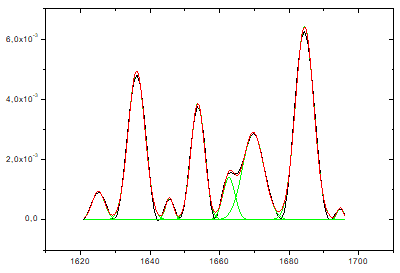

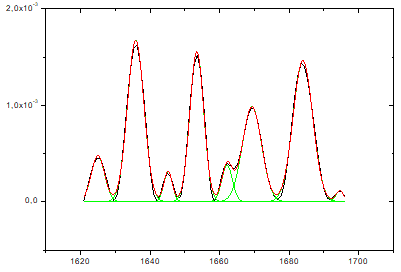

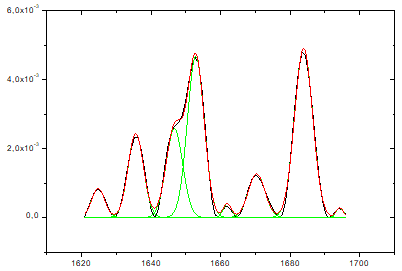

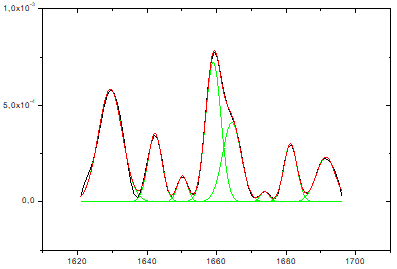


Absorbance

Wavenumber [cm^-1^]

Absorbance

Wavenumber [cm^-1^]

Absorbance

Wavenumber [cm^-1^]

Absorbance

Wavenumber [cm^-1^]


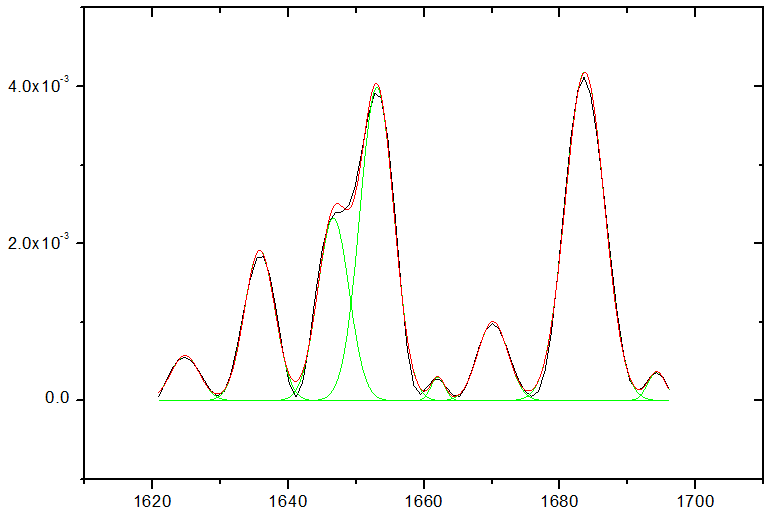


**A B**

**C D**

**E F**

**G H**

Absorbance

Wavenumber [cm^-1^]

Absorbance

Wavenumber [cm^-1^]

Absorbance

Wavenumber [cm^-1^]

Absorbance

Wavenumber [cm^-1^]

**Figure S9:** ATR-FTIR peak analysis for (A) native insulin, (B) insulin amyloid fibrils, insulin aggregates formed in the presence of compound (C) EGCG, binary mixtures (D) GA:EGCG, (E) EGCG:EC, and (F) EGCG:CF; ternary mixtures (G) GA:EGCG:EC, (H) GA: EGCG:CF, (I) EGCG:EC:CF, and (J) GA:EC:CF, and in the presence of quaternary mixture (K) GA:EGCG:EC:CF. All deconvolutions were done in Origin 8.


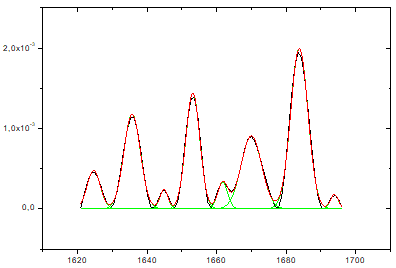

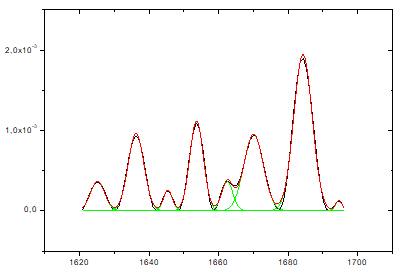

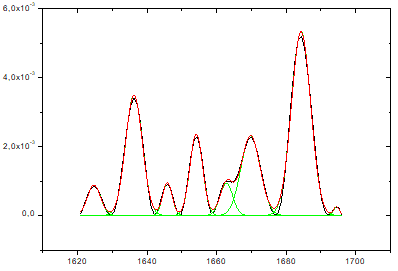


Absorbance

Wavenumber [cm^-1^]

**I J**

**K**

Absorbance

Wavenumber [cm^-1^]

Absorbance

Wavenumber [cm^-1^]

**Table S1:** Secondary structure percentage distributions of samples measured by ATR-FTIR with corresponding peak positions and adjusted R^2^ coefficient representing precision of the deconvolution.

| Secondary structure | β-sheet [%] | random coil [%] | α- + 3_10_-helix [%] | β-turn [%] | antiparallel  β-sheet  [%] | Adjusted R^2^ |
| --- | --- | --- | --- | --- | --- | --- |
| Position of bands  [cm^-1^] | 1623 - 1643 | 1646 - 1650 | 1654 - 1664 | 1666 - 1687 | 1689 - 1698 |  |
| Native insulin | 15.3 | 15.0 | 28.7 | 39.8 | 1.2 | 0.995 |
| Insulin amyloid fibrils | 39.5 | 2.9 | 23.5 | 25.2 | 8.9 | 0.994 |
| + EGCG | 17.0 | 15.1 | 28.6 | 38.6 | 0.7 | 0.993 |
| + GA:EGCG | 21.6 | 1.9 | 25.5 | 50.3 | 0.7 | 0.993 |
| + EGCG:EC | 30.6 | 2.4 | 23.1 | 43.1 | 0.8 | 0.994 |
| + EGCG:CF | 27.0 | 1.6 | 19.9 | 50.8 | 0.7 | 0.994 |
| + GA:EGCG:EC | 26.5 | 4.6 | 16.7 | 51.7 | 0.5 | 0.993 |
| + GA:EGCG:CF | 24.0 | 2.7 | 22.6 | 49.4 | 1.3 | 0.993 |
| + EGCG:EC:CF | 21.0 | 2.3 | 19.9 | 56.0 | 0.8 | 0.994 |
| + EC:GA:CF | 26.1 | 3.3 | 15.6 | 54.5 | 0.5 | 0.993 |
| + GA:EGCG:EC:CF | 24.3 | 1.8 | 22.3 | 50.3 | 1.3 | 0.993 |
